# Impact of SARS-CoV-2 vaccination on CD4^+^ and CD8^+^ T lymphocyte profiles in an HIV-positive and HIV-negative female cohort

**DOI:** 10.1093/labmed/lmag014

**Published:** 2026-04-19

**Authors:** Olive Khaliq, Niren Maharaj, Mikyle David, Ahmad Jassen, Nomakhuwa Tabane, Jagidesa Moodley

**Affiliations:** Department of Paediatrics and Child Health, School of Clinical Medicine, Faculty of Health Sciences, University of the Free State, Bloemfontein, South Africa; Department of Obstetrics and Gynaecology, School of Clinical Medicine, Faculty of Health Sciences, University of the Free State, Bloemfontein, South Africa; Department of Obstetrics and Gynaecology, School of Clinical Medicine, Faculty of Health Sciences, University of the Free State, Bloemfontein, South Africa; Department of Paediatrics and Child Health, School of Clinical Medicine, Faculty of Health Sciences, University of the Free State, Bloemfontein, South Africa; Department of Paediatrics and Child Health, School of Clinical Medicine, Faculty of Health Sciences, University of the Free State, Bloemfontein, South Africa; Department of Obstetrics and Gynaecology, School of Clinical Medicine, Faculty of Health Sciences, Women’s Health and HIV, University of KwaZulu-Natal, Durban, South Africa

**Keywords:** COVID-19, HIV, CD4^+^, CD8^+^

## Abstract

**Introduction:**

The COVID-19 pandemic led to rapid global vaccine deployment, especially among high-risk groups, such as individuals living with HIV. Data are limited, however, on the immunologic effects of SARS-CoV-2 vaccination—specifically, on CD4^+^ and CD8^+^ T lymphocyte levels—in HIV-positive women in South Africa, a population with high HIV prevalence.

**Methods:**

This prospective cross-sectional study included 40 women (aged 14-42 years) admitted to a South African tertiary-care hospital, stratified by HIV and SARS-CoV-2 vaccination status. Flow cytometry (BD Multitest [BD Biosciences]) was used to determine absolute CD4^+^ and CD8^+^ T-cell counts. Data were analyzed with GraphPad Prism, version 8, software (GraphPad Software). The Mann-Whitney *U* test was used for comparisons between 2 independent groups. For comparisons across more than 2 groups, either a 1-way analysis of variance or the Kruskal-Wallis test was applied, with statistically significant results followed by the Dunn multiple comparisons test. Spearman correlation was used to assess relationships between variables. In all cases, statistical significance was defined as *P* < .05

**Results:**

Of the 40 participants, 27 (68%) were HIV positive and 20 (50%) were vaccinated. CD4^+^ T-cell counts were statistically significantly higher in HIV-negative women than in HIV-positive women (*P* = .01), while CD8^+^ levels did not differ significantly (*P* = .41). Vaccination status had no statistically significant impact on CD4^+^ or CD8^+^ counts. The CD4/CD8 ratio was statistically significantly higher in HIV-positive women (*P* = .01), especially among the unvaccinated subgroup (*P* = .002).

**Conclusions:**

SARS-CoV-2 vaccination did not substantially alter CD4^+^ or CD8^+^ T lymphocyte levels, regardless of HIV status.

## Introduction

The COVID-19 pandemic was associated with substantially mortality globally.^1^ SARS-CoV-2 vaccines were developed rapidly and administered globally in an urgent global effort to reduce morbidity and mortality associated with SARS-CoV-2.^1^ According to data published in 2024, a total of 14 billion doses, including boosters, were administered per person globally, with 874.76 million vaccinations in Africa, including 14.8 million vaccinations in South Africa.^2^ Due to the increasing number of deaths that occurred during the pandemic, many vaccine trials were conducted with urgency.^3^ Immunocompromised individuals represented a targeted group who members were vaccinated urgently because their underlying conditions predisposed them to severe COVID-19.^4^ Due to the need for rapid vaccine development and administration, the impact of the vaccines on HIV-infected individuals was not well investigated.^5^

Sub-Saharan Africa had the highest burden of HIV, at 67% in 2021^6^; South Africa is recorded as the leading country in HIV burden, with a prevalence as high as 12.7% in 2024.^7^ In the 2022 Joint United Nations Programme on HIV/AIDS (UNAIDS) data, in eastern and southern Africa, 20.8 million people are listed as living with HIV, of which 500 000 are new infections.^8^ Approximately 62% of women are living with HIV in sub-Saharan Africa, and most are between the ages of 15 and 24 years.^9^ In South Africa, 7.8 million people live with HIV, and 91% are on antiretroviral therapy (ART).^9^ There is a paucity of data, however, on the effect of SARS-CoV-2 vaccination on CD4 and CD8 T cells, particularly in women, who made up the majority (64%) of HIV infections in the South African population in 2021.^10^^,^^11^

In HIV-positive individuals, the number of CD4 T cells decreases as the HIV virus replicates.^12^ Tinago et al^13^ showed that HIV infection results in persistent elevation of terminally differentiated effector memory CD8 T cells and a subsequent decrease in naive and central memory CD8 T cells, leading to a decline in the CD4/CD8 ratio.^13^ To support this hypothesis, another study investigated whether antiretroviral drugs restore the CD4/CD8 ratio to equal that of HIV-negative individuals. The study concluded that the median CD4/CD8 ratio remained decreased compared with HIV-negative individuals due to the persistently high levels of CD8.^14^

The safety and efficacy of SARS-CoV-2 vaccinations have been confirmed in various trials,^3^^,^^4^ but further research is required to understand the effect of the vaccines on immunosuppressed, HIV-infected individuals.^15^ A study in China compared SARS-CoV-2–vaccinated people living with HIV to unvaccinated people with HIV. It found that vaccinated people had a higher CD4/CD8 ratio than unvaccinated people did. An increasing trend was noted as the number of vaccine doses increased.^10^ Interestingly, SARS-CoV-2 vaccination in HIV-positive individuals has been reported to augment CD4 counts to reach a favorable immune response, similar to that of a healthy population, except in cases where the CD4 count is low.^16^

A study in Italy investigated whether the SARS-CoV-2 messenger RNA had an impact on HIV-related immunologic parameters, which included 510 HIV-positive participants. Approximately 81% of the participants received 3 doses of the SARS-CoV-2 vaccine. Their CD4 count increased by 15 cells/mm^3^ after 30 days, while the viral load decreased by −0.11 log_10_.^17^

The effect of the SARS-CoV-2 vaccine on the CD4/CD8 ratio in HIV-positive individuals requires further investigation because limited studies have investigated these cellular dynamics. HIV overburdens South Africa, and the majority of infected people are women older than 15 years of age (20% in women vs 12% in men).^10^ Therefore, this study aimed to investigate the impact of SARS-CoV-2 vaccination on CD4 and CD8 levels in HIV-positive and HIV-negative women in South African.

## Methods

### Study population

A total of 40 women whose babies were admitted to the neonatal unit at a tertiary-care hospital in South Africa were recruited. This study was ethically approved by the Institutional Health Research Ethics Committee (UFS-HSD2022/1456/2609-0004). Informed consent was obtained from all participants who agreed to take part in the study. These women were recruited in 2024 and were between the ages of 14 and 42 years. The study included all women, with or without HIV infection. Their SARS-CoV-2 vaccination status was collected between 2020 and 2022. Previous COVID-19 and the type of SARS-CoV-2 vaccine were also included. All women who declined to participate were excluded.

### Laboratory techniques

A phlebotomist collected all samples from a registered national laboratory. CD4 and CD8 T lymphocytes were measured using the BD FACSLyric Clinical System (BD Biosciences) and processed either by the BD FACSDuet System (BD Biosciences) or manually according to instruction manual provided in Supplementary Material.

### Data analysis

Data analysis was conducted using GraphPad Prism, version 8, software for Windows. Due to the nonparametric distribution of the data, results are presented as medians (IQRs). The Mann-Whitney *U* test was used to assess statistical significance based on absolute CD4 and CD8 counts by vaccination status (vaccinated vs unvaccinated) and HIV status (negative vs positive) as well as the CD4/CD8 ratio by vaccination status (vaccinated vs unvaccinated) and HIV status (negative vs positive). Correlations between groups were evaluated using the Spearman rank correlation coefficient (Spearman ρ). A 1-way analysis of variance test and a Kruskal-Wallis test in combination with the Dunn multiple comparison post hoc test was used. *P* < .05 was considered statistically significant.

## Results

The study population was women (*n* = 40) with a median (IQR) age of 24 (14-42) years. Of these 40 women, 27 were HIV positive (on ART) and 13 were HIV negative, yielding a rate of 68%. Thirty of the 40 women tested positive for COVID-19 during the study period. Before the study, 3 women had had COVID-19. Twenty women had received the SARS-CoV-2 vaccination in 2021 and 2022; 12 of the vaccinated women received the Johnson & Johnson vaccine and 8 received the Pfizer vaccine. Of the 8 women who received the Pfizer vaccine, only 2 received their second dose. Of the 12 who received the Johnson & Johnson vaccine, 1 received a booster shot.

**Figure 1:**
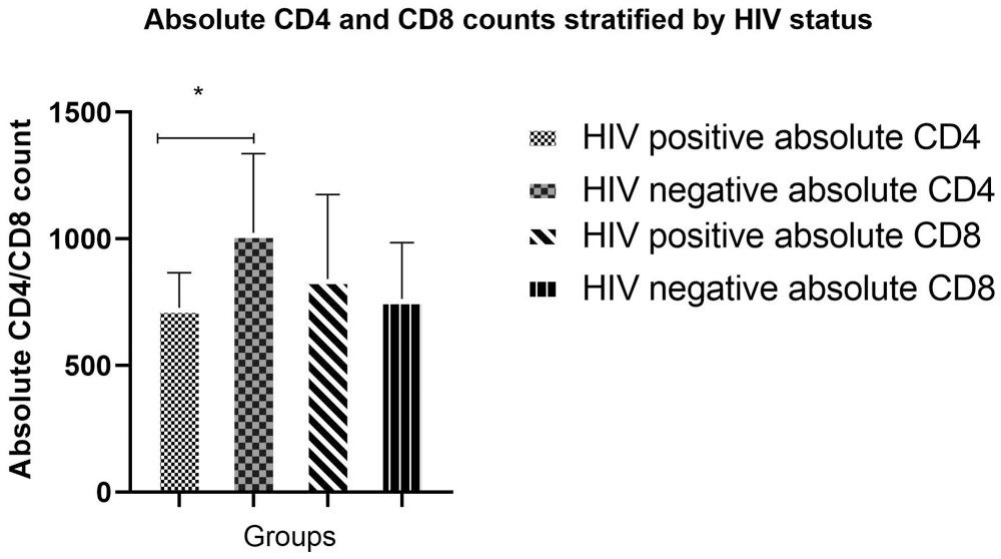
Histogram illustrating the absolute CD4 and CD8 concentrations, stratified by HIV status. *P < 0.05

The absolute concentrations of CD4 and CD8 T cells were compared between HIV-negative and HIV-positive SARS-CoV-2–vaccinated individuals. A statistically significant difference (*P* = .01) was observed in the absolute CD4 concentrations between the 2 groups (Table 1, Figure 1). HIV-negative participants demonstrated a statistically significantly higher median (IQR) range of CD4 concentration (876 [650-1102] × 10 cells/mm^3^) compared with their HIV-positive counterparts (*P* = .01). In contrast, the absolute CD8 concentrations did not differ significantly between HIV-negative and HIV-positive individuals (median [IQR], 885 [583-1238] × 10 cells/mm^3^; *P* = .41) (Table 1, Figure 1).

**Table 1:** Absolute CD4 and CD8 concentrations, by HIV status (*n* = 40).

Absolute CD4 and CD8 concentration	HIV negative vs HIV positive, median (IQR), ×10 cells/mm^3^	*P* value
CD4	876 (650-1102) 1024 for HIV negative vs 728 for HIV positive	.01***
CD8	885 (583-1238) 846 for HIV negative vs 924 for HIV positive	.41

*** *P* < .05.

**Figure 2:**
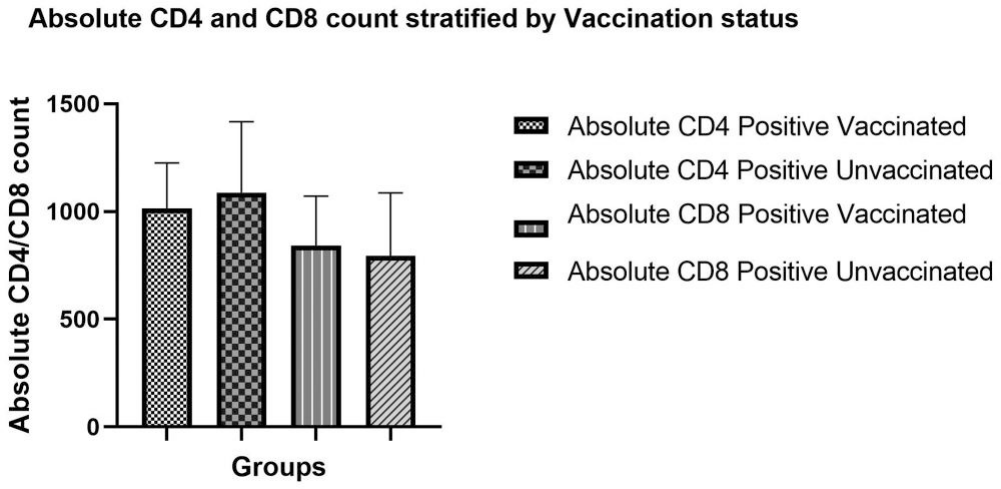
Histogram illustrating the absolute CD4 and absolute CD8 concentrations and vaccination status.

Our cohort consisted of 20 vaccinated and 20 unvaccinated women. The absolute concentrations of CD4 and CD8 T cells were compared in the vaccinated cohort. No statistically significant differences were observed in the absolute CD4 and CD8 concentrations in the vaccinated group (median, 885 [719-1107] × 10 cells/mm^3^; *P* = .07) (Table 2, Figure 2). Similarly, despite an increase in the median (IQR), no statistically significant differences were noted in the CD4 and CD8 absolute concentrations in the unvaccinated group (median [IQR], 980 [740-1189] ×10 cells/mm^3^; *P* = .82) (Table 2; Figure 2).

**Table 2:** Absolute CD4 and CD8 concentrations, by SARS-CoV-2 vaccination status.

Absolute CD4 and CD8 concentration	Vaccinated vs unvaccinated, median (IQR), cells/mm^3^	***P*** **value**
Vaccinated CD4 and CD8 (*n* = 20)	885 (719-1107) 992 for CD4 vs 778 for CD8	.07
Unvaccinated CD4 and CD8 (*n* = 20)	980 (740-1189) 998 for CD4 vs 963 for CD8	.82
Absolute CD4 levels of vaccinated HIV-positive women (*n* = 17) vs unvaccinated HIV-positive women (*n* = 10)	1015 (801-1226) for HIV-positive vaccinated women vs 1088 (969-1417) for HIV-positive unvaccinated women	.39
Absolute CD8 levels of vaccinated HIV-positive women (*n* = 17) vs unvaccinated HIV-positive women (*n* = 10)	842 (726-1072) for HIV-positive vaccinated women vs 795 (659-1087) for HIV-positive unvaccinated women	.68

**Figure 3:**
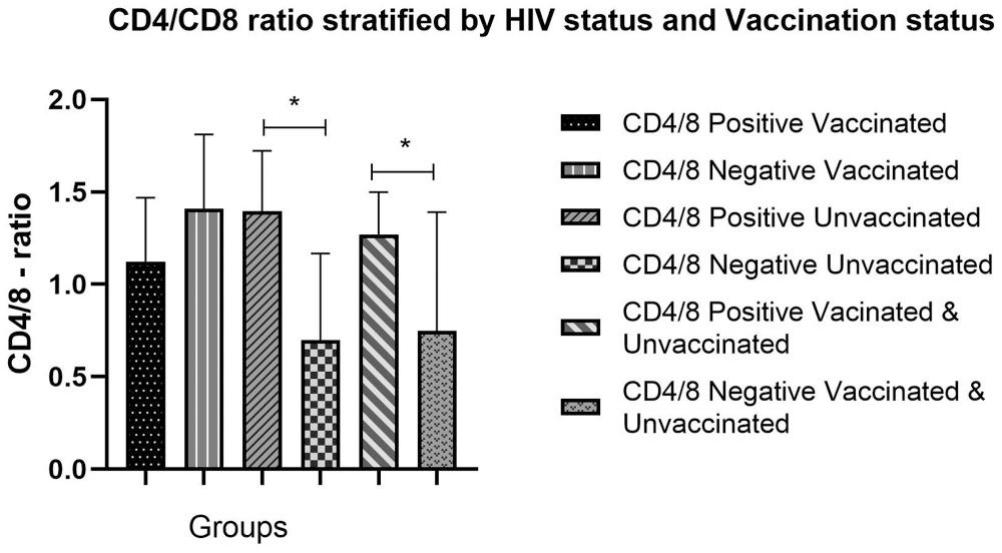
Histogram illustrating the CD4/CD8 ratio, stratified by HIV and vaccination status. *P < 0.05

We evaluated the CD4/CD8 ratio across different groups, stratified by HIV status and vaccination status. Among HIV-positive women, there was no statistically significant difference in the CD4/CD8 ratio between vaccinated and unvaccinated participants (median [IQR], 1.26 [1.04-1.59]; *P* = .13). Similarly, in the HIV-negative cohort, vaccination status did not substantially affect the CD4/CD8 ratio (median [IQR], 1.06 [0.62-1.49]; *P* = .12). When comparing HIV-positive and HIV-negative women, the CD4/CD8 ratio was statistically significantly higher in the HIV-positive group (median [IQR], 1.27 [0.91-1.50]) than in the HIV-negative group (median [IQR], 0.75 [0.56-1.39]), with an overall HIV-positive vs HIV-negative median (IQR) of 1.01 (0.74-1.45) (*P* = .01) (Table 3, Figure 3). Similarly, further subgroup analysis showed a statistically significantly higher CD4/CD8 ratio in the unvaccinated HIV-positive vs HIV-negative group (median [IQR], 1.05 [0.86-1.45]; *P* = .002), but no statistically significant difference was found in the vaccinated HIV-positive and HIV-negative groups (median [IQR], 1.27 [0.80-1.64]; *P* = .82) (Table 3; Figure 3).

**Table 3:** CD4/CD8 ratio, by HIV status and SARS-CoV-2 vaccination status.

HIV status vs vaccination status	**CD4/CD8 ratio: reference range (1.0-2.1), median (IQR)**	***P*** **value**
HIV positive (vaccinated vs unvaccinated) (*n* = 27)	1.26 (1.04-1.59)	.13
HIV negative (vaccinated vs unvaccinated) (*n* = 13)	1.06 (0.62-1.49)	.12
HIV positive vs HIV negative (*n* = 40)	1.01 (0.74-1.45)	.01*
HIV negative vs HIV positive (unvaccinated) (*n* = 20)	1.05 (0.86-1.45)	.002**
HIV negative vs HIV positive (vaccinated) (*n* = 20)	1.27 (0.80-1.64)	.82

**P < .005, *P < 0.05.

When stratified by SARS-CoV-2 infection status, individuals who had COVID-19 during the study period (*n* = 37) exhibited higher CD4 concentrations (median [IQR], 999 [769-1221] × 10 cells/mm³) than did those who had had COVID-19 before the study period (*n* = 3; median [IQR], 836 [533-1075] ×10 cells/mm^3^) (Table 4), although this difference did not reach statistical significance (*P* = .34). Combined data from all participants yielded a median (IQR) CD4 concentration of 918 [651-1148] × 10 cells/mm^3^) (Table 4).

**Table 4:** CD4/CD8 concentrations, by COVID-19 status.

Absolute CD4/CD8 concentration	Positive during the study period, median (IQR), cells/mm^3^ (*n* = 37)	Negative during the study period, median (IQR), cells/mm^3^ (*n* = 3)	Positive vs negative during the study period, median (IQR), cells/mm^3^ (*n* = 40)	***P*** **value**
CD4	999 (769-1221)	836 (533-1075)	918 (651-1148)	.34
CD8	884 (704-1124)	750 (644-763)	817 (674-944)	.21

For CD8 concentrations, values were relatively consistent across groups (median [IQR], 884 [704-1124] ×10 cells/mm^3^) during the study period, with a median (IQR) of 750 (644-763) ×10 cells/mm^3^ before the study (*P* = .21). The combined median (IQR) CD8 concentration was 817 (674-944) × 10 cells/mm^3^) (Table 4).

Across the study period (2021-2022), there were no statistically significant differences in the absolute CD4 or CD8 T-cell concentrations among vaccinated participants. Median (IQR) CD4 concentrations were 1015 (773-1209) ×10 cells/mm^3^ in 2022 and 999 (842-1241) ×10 cells/mm^3^ in 2021 (*P* = .94). Similarly, CD8 concentrations remained comparable between years, with a median (IQR) of 842 (749-1115) ×10 cells/mm^3^ in 2022 and 796 (681-1206) ×10 cells/mm^3^ in 2021 (*P* = .71) (Table 5).

**Table 5:** CD4 and CD8 absolute concentration, by year of SARS-CoV-2 vaccination.

Absolute CD4/CD8 concentration	2022 SARS-CoV-2 vaccination, median (IQR), cells/mm^3^ (*n* = 11)	2021 SARS-CoV-2 vaccination, median (IQR), cells/mm^3^ (*n* = 9)	***P*** **value**
CD4	1015 (773-1209)	999 (842-1241)	.94
CD8	842 (749-1115)	796 (681-1206)	.71

**Figure 4:**
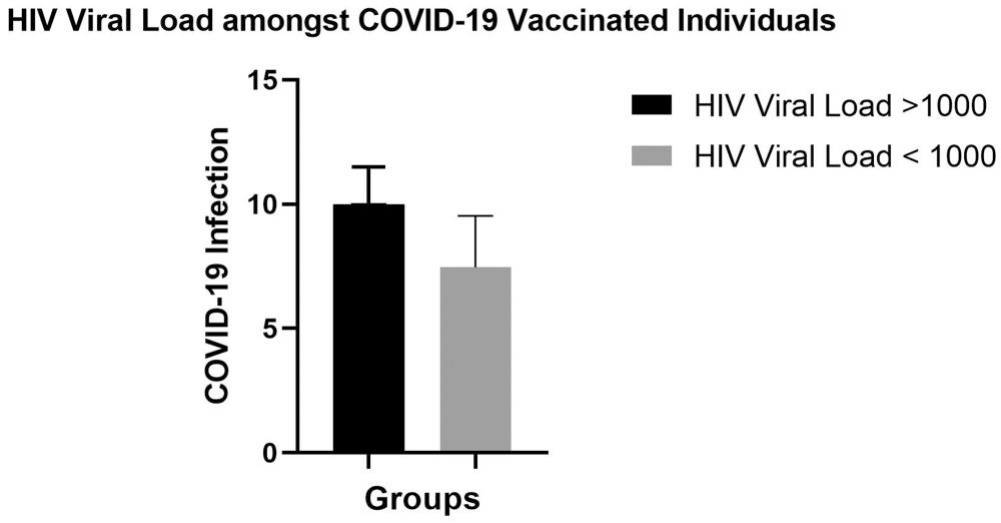
Histogram illustrating the HIV viral load and SARS-CoV-2 infection among vaccinated individuals with a viral load >1000 copies and <1000 copies (*n* = 13).

An analysis was performed between the viral load (>1000 copies and <1000 copies) and SARS-CoV-2 infection among vaccinated individuals. There was no statistically significant difference between the viral load and SARS-CoV-2 infection among the vaccinated group (median [IQR], 8.73 [4.31-9.76]; *P* = .93) (Figure 4).

## Discussion

This study showed a statistically significant difference in absolute CD4 levels between HIV-positive women on ART and HIV-negative women (*P* = .01). This finding was expected as HIV viral replication depletes CD4 levels. Absolute CD4 levels are used as a biomarker for the progression of HIV, the effectiveness of treatment, and the outcome.^18^^,^^19^ These cells are a measure of the human body’s immune response to infection.^18^ HIV penetrates these cells, allowing bacterial and viral pathogen invasion and replication and leading to an increased risk of HIV-related complications.^20^ Antiretroviral therapy elevates CD4 levels and reduces HIV viral load to improve clinical outcomes in HIV-positive individuals.^20^ Our findings align with data from Urassa et al,^21^ which showed a decrease in CD4 levels in HIV-positive individuals compared with HIV-negative individuals.

No substantial difference was found in absolute CD8 levels in HIV-positive vs HIV-negative women in this study. In addition, there was no correlation between the HIV viral load and SARS-CoV-2 infection. These findings highlight the expected reduction in CD4 T cells due to HIV infection while suggesting that CD8 T-cell levels are relatively preserved, irrespective of HIV status (Table 1). During HIV viral invasion, CD4 T lymphocytes are destroyed; however, CD8 T lymphocytes increase in HIV-positive individuals,^22^ which explains the nonsignificant difference noted in HIV-positive vs HIV-negative women in this study. Generally, CD8 levels remain elevated in HIV-positive individuals until the end stage of chronic HIV, where all T cells are destroyed.^23^ Furthermore, ART is reported to increase CD4 T lymphocytes, with little or no effect noted in CD8 levels, highlighting the role of CD4 T lymphocytes as the key T cells used to monitor HIV progression and management.^24^ Moreover, other studies associated persistent CD8 T lymphocytes with clinical manifestations unrelated to AIDS, such as cardiovascular disease and malignancies, despite improved CD4 T lymphocytes.^24^^,^^25^

This study found no statistically significant difference in absolute CD4 and CD8 T lymphocytes in SARS-CoV-2–vaccinated and SARS-CoV-2–unvaccinated HIV-positive and HIV-negative women. Furthermore, no statistically significant difference was found in HIV-positive women regardless of SARS-CoV-2 vaccination status, with a slight increase of CD4 levels noted in the unvaccinated group (Table 2). These findings differ from those of Walsh et al,^25^ however, who suggested that SARS-CoV-2 vaccination increased immune response and activated Th1 CD4^+^ and CD8^+^ T cells in male and nonpregnant female individuals.^25^ In addition, another study from Italy reported elevated CD4 levels 30 days after SARS-CoV-2 vaccination.^17^ The difference in findings may also be because the participants in our study received their SARS-CoV-2 vaccinations in 2020, 2021, and 2022. The CD4 and CD8 levels in this study were measured between 2 and 4 years after vaccination.^17^ This study also found no statistically significant difference in CD4/CD8 lymphocytes in women vaccinated in 2021 compared with women vaccinated in 2022. This hypothesis is supported by Li et al,^10^ who reported an increase in CD4/CD8 ratio within 2 months of receiving the vaccine but suggested that the immune response the vaccine generates declines over time and returns to levels seen in unvaccinated HIV-positive individuals.^10^

The CD4/CD8 ratio was statistically significantly higher in HIV-positive women than in HIV-negative women, regardless of their SARS-CoV-2 vaccination status (*P* = .01). Furthermore, the results showed a statistically significantly higher CD4/CD8 ratio in HIV-positive unvaccinated women than in HIV-negative unvaccinated women (*P* = .002). Contrasting results were reported by Gras et al,^14^ who suggested that the CD4/CD8 ratio declines in HIV-positive individuals compared with HIV-negative individuals, even after ART, due to the persistent CD8 elevation observed in HIV-positive individuals. Interestingly, a comparison between SARS-CoV-2–vaccinated HIV-positive women and SARS-CoV-2–vaccinated HIV-negative women showed no statistical difference. As suggested by Li et al,^10^ the insignificant finding in the CD4/CD8 ratio in SARS-CoV-2–vaccinated HIV-positive vs HIV-negative women may be due to an increase in the CD4/CD8 ratio after vaccination. In addition, the CD4/CD8 ratio in HIV-positive participants is reduced over time to levels that are still higher than before the vaccine was administered.^10^

### Study limitations and strengths

The relatively small sample size in this study may limit the generalizability of our findings. In addition, the collection of data 2 to 4 years after the administration of the SARS-CoV-2 vaccine may not reflect immediate or short-term immunologic changes. The inclusion of both HIV-positive and HIV-negative participants, however, facilitated comparative insights into the immune profiles of women in a real-world setting.

## Conclusion

SARS-CoV-2 vaccination did not substantially alter CD4^+^ or CD8^+^ T lymphocyte profiles in either HIV-positive or HIV-negative women. The observed elevation in absolute CD4 counts among HIV-positive individuals is consistent with existing trends in this population. Similarly, CD8^+^ counts remained stable across both groups following vaccination. These findings indicate that that SARS-CoV-2 vaccine has minimal to no effect on CD4 and CD8 T lymphocyte dynamics, suggesting that it may not substantially affect the adaptive immune response in women, regardless of HIV status.

## Supplementary Material

lmag014_Supplementary_Data

## Data Availability

All data pertaining to this study are available from the corresponding author upon request.

## Supplementary Material: Instructions manual

BD Multitest CD3/CD8/CD45/CD4 is a four-colour immunofluorescent reagent for use with the BD FACS Lyric and processed either by the BD FACS Duet or manually. The percentages and absolute counts of mature human T lymphocytes (CD3+), suppressor/cytotoxic T lymphocyte subsets (CD3+CD8+), and helper/inducer T lymphocyte subsets (CD3+CD4+) are measured employing flow cytometry in erythrocyte-lysed whole blood.

Determining percentages or counts of CD4 lymphocytes is used for monitoring HIV-infected individuals, who typically exhibit a steady decrease in CD4 counts as the infection progresses. The relevant percentage of the CD8 subset is elevated in many patients with AIDS. The enumeration of the CD4 and CD8 lymphocytes also plays an important role in the monitoring of ARV treatment in HIV positive patients.

When reagent is added to whole blood, the fluorochrome-labelled antibodies in the reagent bind specifically to leukocyte surface antigens. During acquisition, the cells travel past the flow cytometer laser beams and scatter the light. The stained cells fluoresce. The scatter and fluorescence signals, detected by the instrument, provide information about the cell’s size (FSC), internal complexity (SSC) and relative fluorescent intensity. The reagents employ fluorescent triggering, which allows direct fluorescence gating of the lymphocyte populations.

A known volume of specimen is stained in the BD Trucount tubes, releasing a known number of fluorescent beads. During analysis, the absolute number of positive cells in the sample can be determined by comparing cellular events to bead events.
