# Supplementary material for: Impact of SARS-CoV-2 vaccination on CD4+ and CD8+ T lymphocyte profiles in an HIV-positive and HIV-negative female cohort
Source: Lab Med. 2026 Apr 19;57(3):lmag014. doi: 10.1093/labmed/lmag014 (PMC13092133; doi:10.1093/labmed/lmag014)
Supplement: lmag014_Supplementary_Data [file lmag014_supplementary_data.docx]

**Supplementary Material: Instructions manual**

BD Multitest CD3/CD8/CD45/CD4 is a four-colour immunofluorescent reagent for use with the BD FACS Lyric and processed either by the BD FACS Duet or manually. The percentages and absolute counts of mature human T lymphocytes (CD3+), suppressor/cytotoxic T lymphocyte subsets (CD3 + CD8+), and helper/inducer T lymphocyte subsets (CD3 + CD4+) are measured employing flow cytometry in erythrocyte-lysed whole blood.

Determining percentages or counts of CD4 lymphocytes is used for monitoring HIV-infected individuals, who typically exhibit a steady decrease in CD4 counts as the infection progresses. The relevant percentage of the CD8 subset is elevated in many patients with AIDS. The enumeration of the CD4 and CD8 lymphocytes also plays an important role in the monitoring of ARV treatment in HIV positive patients.

When reagent is added to whole blood, the fluorochrome-labelled antibodies in the reagent bind specifically to leukocyte surface antigens. During acquisition, the cells travel past the flow cytometer laser beams and scatter the light. The stained cells fluoresce. The scatter and fluorescence signals, detected by the instrument, provide information about the cell’s size (FSC), internal complexity (SSC) and relative fluorescent intensity. The reagents employ fluorescent triggering, which allows direct fluorescence gating of the lymphocyte populations.

A known volume of specimen is stained in the BD Trucount tubes, releasing a known number of fluorescent beads. During analysis, the absolute number of positive cells in the sample can be determined by comparing cellular events to bead events.
